# The efficacy and safety of combined immune checkpoint inhibitors (nivolumab plus ipilimumab): a systematic review and meta-analysis

**DOI:** 10.1186/s12957-020-01933-5

**Published:** 2020-07-03

**Authors:** Jingjie Chen, Shengnan Li, Qigu Yao, Nannan Du, Xiaojun Fu, Yuanmei Lou, Mengru Wang, Feiyan Mao, Danyi Mao, Parikshit Asutosh Khadaroo, Yingying Tang

**Affiliations:** 1 Department of General Surgery, HwaMei Hospital, University of Chinese Academy of Sciences, Ningbo, Zhejiang China; 2 Ningbo Institute of Life and Health Industry, University of Chinese Academy of Sciences, Ningbo, Zhejiang China; 3 Key Laboratory of Diagnosis and Treatment of Digestive System Tumors of Zhejiang Province, Ningbo, Zhejiang China; 4 The Second Clinical Medical College, Zhejiang Chinese Medical University, Hangzhou, Zhejiang China; 5 State Key Laboratory for Diagnosis and Treatment of Infectious Diseases, the First Affiliated Hospital, College of Medicine, Zhejiang University, 79 Qingchun Rd, Hangzhou City, 310003 China; 6 The Third Clinical Medical College, Zhejiang Chinese Medical University, Hangzhou, Zhejiang China; 7 Medical College of Kaifeng University, Kaifeng, Henan China; 8 Basic Medical College, Zhejiang Chinese Medical University, Hangzhou, Zhejiang China; 9 Monash University School of Medicine, Nursing and Health Sciences, Melbourne, Australia; 10 Department of Radiotherapy and Chemotherapy, HwaMei Hospital, University of Chinese Academy of Sciences, Northwest Street 41, Haishu District, Ningbo, 315010 Zhejiang China

**Keywords:** Nivolumab, Ipilimumab, Tumor response, Adverse events, Meta-analysis

## Abstract

**Background:**

Currently, nivolumab and ipilimumab are the most widely used immune checkpoint inhibitors. We performed a meta-analysis to evaluate the efficacy and treatment-related adverse events (TRAEs) of nivolumab plus ipilimumab therapy in cancer treatment.

**Methods:**

We examined data from PubMed, Web of Science, EBSCO, and Cochrane Library. Eleven articles fulfilled our criteria, which we divided into 3 groups: nivolumab plus ipilimumab versus nivolumab (the dose used for monotherapy is 3 mg/kg), nivolumab plus ipilimumab versus ipilimumab (the dose used for monotherapy is 3 mg/kg), and nivolumab 1 mg/kg plus ipilimumab 3 mg/kg (N1I3) versus nivolumab 3 mg/kg plus ipilimumab 1 mg/kg (N3I1). We measured the complete response (CR), partial response (PR), objective response rate (ORR), and TRAEs in any grade and grade 3 or higher.

**Results:**

The overall effect estimate favored the combined immunotherapy group in terms of the ORR (RR: 1.40, *p* < 0.001) and PR (RR: 1.50, *p* < 0.001) than nivolumab alone. Compared with ipilimumab alone, the combined immunotherapy group had better CR (RR: 4.89, *p* < 0.001), PR (RR: 2.75, *p* < 0.001), and ORR (RR: 3.31, *p* < 0.001). Finally, N1I3 showed better PR (RR: 1.35, *p* = 0.006) and ORR (RR: 1.21, *p* = 0.03) than N3I1. The incidence of any TRAEs was similar between both groups (RR: 1.05, *p* = 0.06). However, the incidence of serious adverse events (grade 3 or higher) was lower in group N3I1 than group N1I3 (RR: 1.51, *p* < 0.001).

**Conclusion:**

This meta-analysis showed that the curative effect of nivolumab plus ipilimumab was better than that of nivolumab or ipilimumab monotherapy. In the combined immunotherapy group, N1I3 was more effective than N3I1. Although the side effects were slightly increased in N1I3 group, overall safety was acceptable.

## Background

Cytotoxic T lymphocyte-associated protein 4 (CTLA-4) is a receptor on the surface of activated T cells [1]. It mainly acts by binding B7 ligand on antigen-presenting cells (APCs). The protein competes with the cluster of differentiation 28 (CD28) receptor for B7 ligand. During T cell activation, CD28 receptors on T cells bind to B7 ligand on antigen-presenting cells (APCs) and provide the essential second activation signal for T cells [2–4]. Programmed cell death protein 1 (PD-1) is a cell-surface receptor commonly found in T cells, B cells, and NK cells [5]. By inhibiting the phosphatidylinositol 3-kinase (PI3K) pathway, PD-1 signaling inhibits the activation of the cell survival factor Bcl-xL and the expression of transcription factors such as GATA-3, T-bet, and Eomes [6], that regulate T cell functions. The CTLA-4 and PD-1, two common immune checkpoint inhibitors (ICIs) on activated T cells, are the most reliable targets for cancer treatment. Up to now, seven drugs targeting CTLA-4/PD-1 have been approved for treatment of different types of cancer, including melanomas [7], lung [8], breast [9], cervical [10], and liver cancer [11].

In clinical studies, CTLA-4 and PD-1 monotherapy inhibitors have shown impressive, lasting effects that have significantly prolong the survival of responsive patients [12, 13]. For example, colorectal cancer (CRC) is the third most common cancer in the USA. Despite advances in chemotherapy, survival rates in patients with metastatic CRC remains low. However, CRC patients treated with immune checkpoint inhibitors showed better response [14]. Hepatectomy is an important method to treat liver cancer, but up to 70% of patients may have a recurrence of liver diseases within 5 years, even after receiving treatment for hepatocellular carcinoma (HCC) at an early stage. However, immunotherapy has recently been shown to be effective against HCC, marking a milestone in the history of this intractable disease [15]. The efficacy of immune-monotherapy is limited by low response rates, with only a small proportion of patients responding to treatment [13]. Rotte et al. [16] reported that drugs when administered as monotherapy had a manageable safety profile, but more than 50% of patients failed to respond to treatment. While the results of monotherapy treatments are not satisfactory, there is increasing emphasis on combination treatments in an effort to increase response rates to treatment. Combination of CTLA-4 and PD-1 blockers was then evaluated to increase the response rates in patients, and the combination of nivolumab plus ipilimumab showed significantly enhance efficacy in metastatic melanoma patients. The combination group has demonstrated numerically higher response rates and improved long-term clinical benefit relative to anti-PD-1/PD-L1 or anti-CTLA-4 monotherapy [14]. Durable responses and encouraging survival have been demonstrated with immune checkpoint inhibitors in small-cell lung cancer (SCLC), and the results showed that nivolumab plus ipilimumab appeared to provide a greater clinical benefit than nivolumab monotherapy in the high tumor mutational burden tertile [17]. Combination of immunotherapies is one of the most promising new methods. Presently, nivolumab and ipilimumab are the most widely used immune checkpoint inhibitors against cancer [18]. This meta-analysis aimed at investigating the role of nivolumab plus ipilimumab therapy in cancer treatment.

## Materials and methods

### Search and selection

We did a meta-analysis of relevant articles, published before by June 2019. We searched through four electronic databases; PubMed, Web of Science, EBSCO, and Cochrane Library for data with relevant clinical trials, based on these keywords: (nivolumab or PD-1 or programmed death 1) and (ipilimumab or CTLA-4 or cytotoxic T-lymphocyte-associated protein 4).

### Inclusion and exclusion criteria

Studies in the selected articles were to meet four criteria: (1) participants: solid tumor patients receiving combined ICIs (nivolumab plus ipilimumab); (2) intervention: combined ICIs including nivolumab and ipilimumab simultaneously; (3) comparisons: nivolumab or ipilimumab alone; (4) outcomes: objective response rate (ORR), partial response rate (PR), complete response (CR), and treatment-related adverse events (TRAEs), otherwise they were excluded. And these studies would be excluded if they were (1) not included in the combination of nivolumab and ipilimumab; (2) non-prospective clinical trials; (3) included other treatments; (4) single-arm study; (5) not relevant outcome.

### Data extraction

We focused on trial phases, tumor types, the number and characteristics of participants, the anti-tumor agents, dosage, and frequency of drug administration with a keen interest on the prognoses, specifically the ORR, CR, and PR. The curative effects were assessed using the Response Evaluation Criteria in Solid Tumors (RECIST). Incidence of TRAEs, including any grade and grade 3 or higher was also evaluated, based on the National Cancer Institute’s Common Terminology Criteria for Adverse Events (CTCAE).

### Quality assessment

We evaluated the quality of data in the relevant articles using the Cochrane Collaboration tool based on the following domains: allocation concealment, masking of outcome assessors, blinding of participants, incomplete follow-up, and selective outcome reporting.

### Statistical analysis

Statistical analyses were done by the Review Manager software (RevMan5.3), at 95% confidence intervals (CI). Subgroup analysis was done based on the intervention: nivolumab plus ipilimumab versus nivolumab alone, nivolumab plus ipilimumab versus ipilimumab alone, nivolumab 1 mg/kg plus ipilimumab 3 mg/kg (N1I3) versus nivolumab 3 mg/kg plus ipilimumab 1 mg/kg (N3I1). All analyses (ORR, CR, PR, and all-grade and serious grade TRAEs) were performed based on the fixed/random-effect. For meta-analysis, we used risk ratios (RR) to compare with dichotomous variables. Heterogeneity in the meta-analysis results was done by the *I*-square (*I*^2^) test. Statistically significant was measured at *P* values of 0.05.

## Results

### Literature search

We identified 4361 studies in the literature search. It then removed 2133 duplicates and further 2228 after careful evaluation. Although 56 articles met the inclusion criteria, 45 were removed besides qualifying for meta-analysis for a number of reasons. Among them, 16 articles were excluded because they did not include the combination of nivolumab and ipilimumab; 11 articles were non-prospective clinical trials; 7 articles were excluded because they included other treatments; 6 articles were single-arm studies; 5 articles were excluded because of irrelevant outcome. In the end, 11 articles [19–29] were qualified for the meta-analysis. The specific reasons are shown in Supplementary Figure 1.

### Study characteristics

The characteristics of each study are shown in Supplementary Table 1. The 11 clinical trials included 2484 patients. Of these 879 received nivolumab 1 mg/kg plus ipilimumab 3 mg/kg (N1I3), 560 received nivolumab 3 mg/kg plus ipilimumab 1 mg/kg (N3I1), when combined, nivolumab is generally used 3 mg/kg, ipilimumab used 1 mg/kg or nivolumab used 1 mg/kg, ipilimumab used 3 mg/kg. Six hundred eighty-eight received nivolumab at a recommended dose of 3 mg/kg monotherapy, while 357 were put on ipilimumab at a recommended dose of 3 mg/kg monotherapy. The selected studies had varied cases, from melanoma, metastatic urothelial carcinoma, small cell lung cancer (SCLC), esophagogastric cancer (EGC), malignant pleural mesothelioma (MPM), renal cell carcinoma (RCC), sarcoma, glioblastoma. There were two phase I clinical trials, two phase I-II clinical trials, four phase II clinical trials, two phase III clinical trials, and one phase III-IV clinical trials.

### Nivolumab plus ipilimumab versus ipilimumab alone

**Fig. 1:**
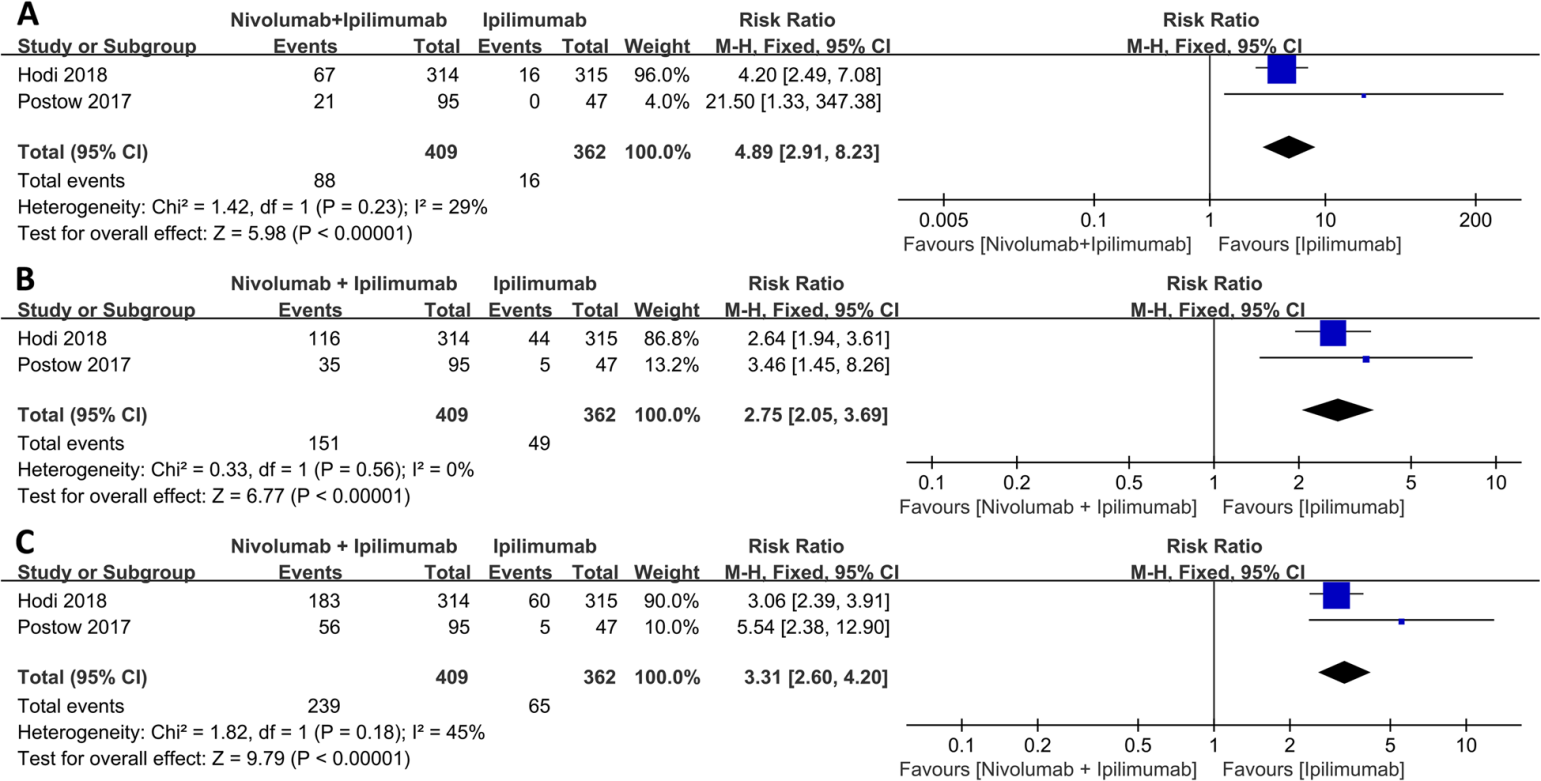
Forest plot of the overall effect between nivolumab combined with ipilimumab and ipilimumab alone. **a** Complete response (CR). **b** Partial response (PR). **c** Objective response rate (ORR)

**Fig. 2:**
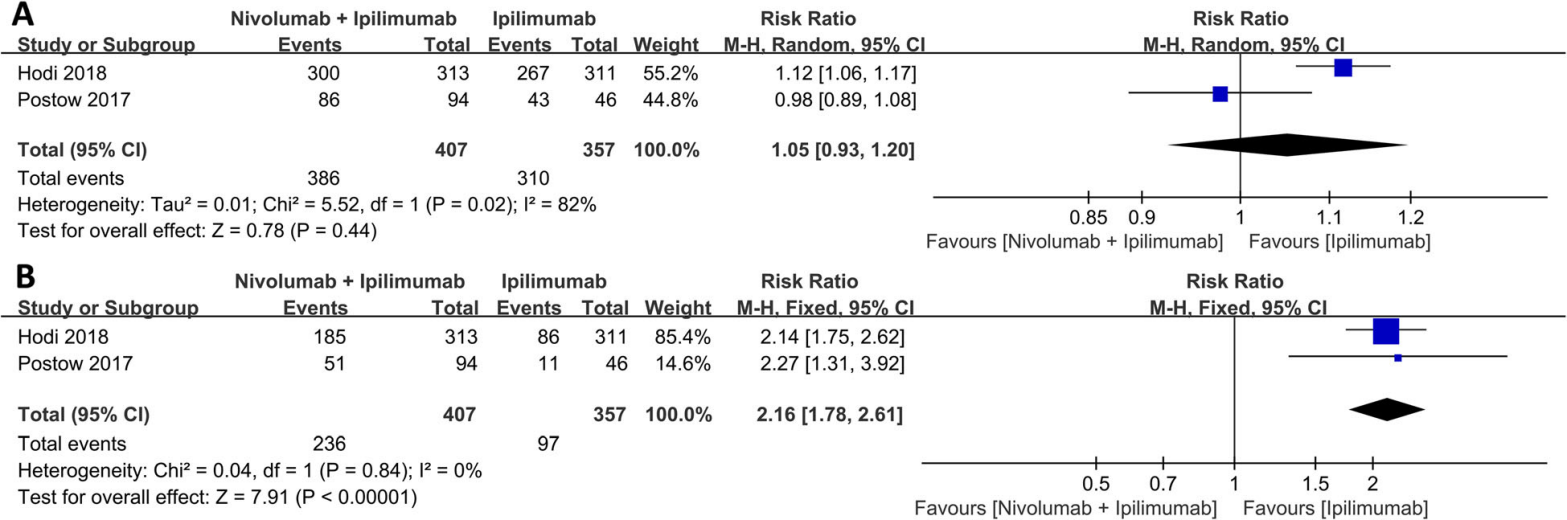
Forest plot of the adverse events between nivolumab combined with ipilimumab and ipilimumab alone. **a** Any grade TRAEs. **b** Grade 3 or higher TRAEs

Compared with ipilimumab alone, nivolumab plus ipilimumab synergy caused a greater effect under CR (RR: 4.89, 95% CI: 2.91–8.23, *p* < 0.001), PR (RR: 2.75, 95% CI: 2.05–3.69, *p* < 0.001), and ORR (RR: 3.31, 95% CI: 2.60–4.20, *p* < 0.001) as shown in Fig. 1. Although the incidence of any TRAEs was similar between the two groups (RR: 1.05, *p* = 0.44), ipilimumab monotherapy resulted in less serious cases, (grade 3 or higher) than nivolumab plus ipilimumab group (RR: 2.16, 95% CI: 1.78–2.61, *p* < 0.001) as shown in Fig. 2.

### Nivolumab plus ipilimumab versus nivolumab alone

**Fig. 3:**
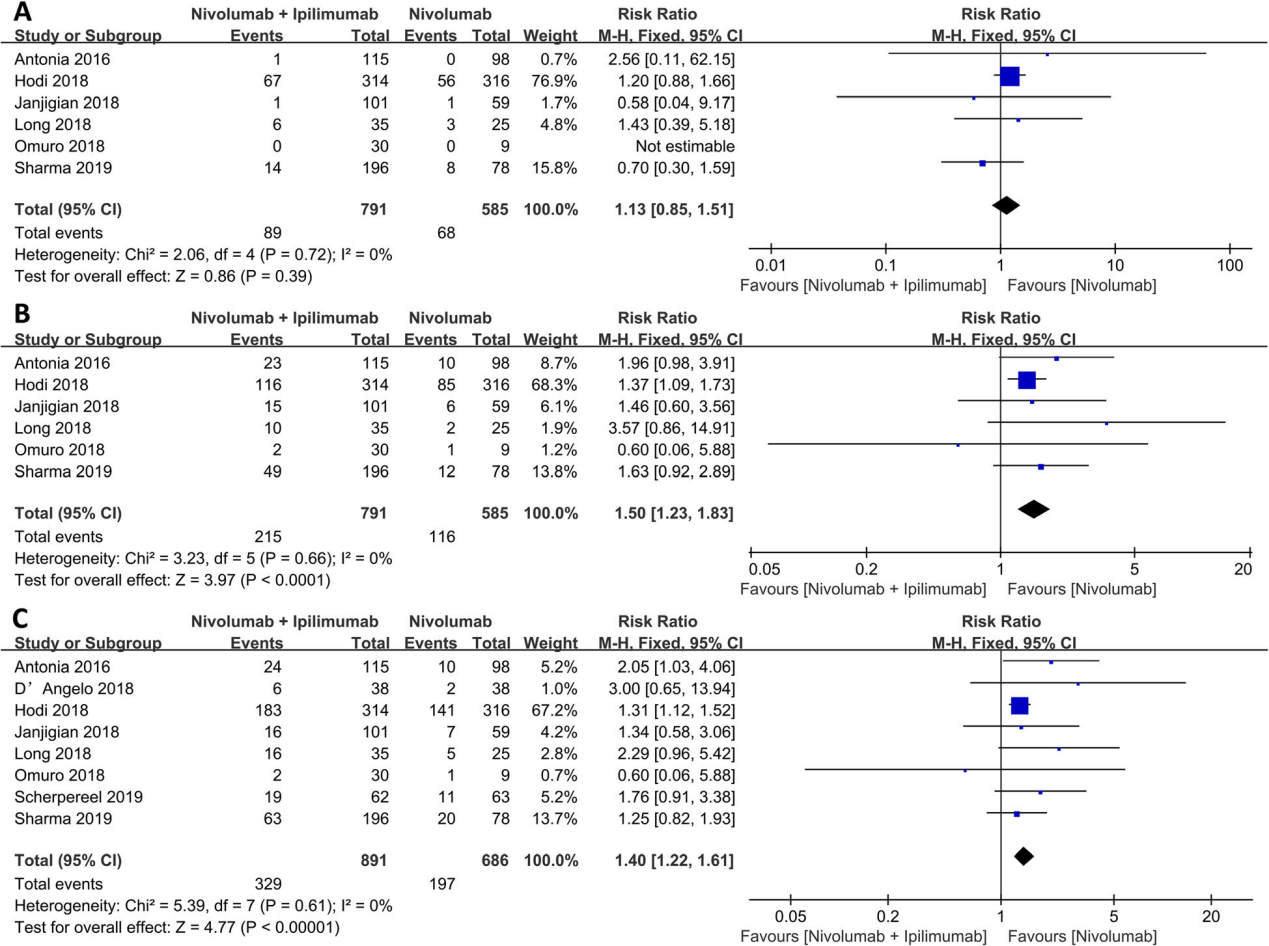
Forest plot of the overall effect between nivolumab-ipilimumab combined therapy and nivolumab monotherapy. **a** Complete response (CR). **b** Partial response (PR). **c** Objective response rate (ORR)

Overall, nivolumab plus ipilimumab group showed better ORR (RR: 1.40, 95% CI: 1.22–1.61, *p* < 0.001) and PR (RR: 1.50, 95% CI: 1.23–1.83, *p* < 0.001) than nivolumab alone; however, there was no statistically significant difference in the CR (RR: 1.13, *p* = 0.39) between the two as shown in Fig. 3.

**Fig. 4:**
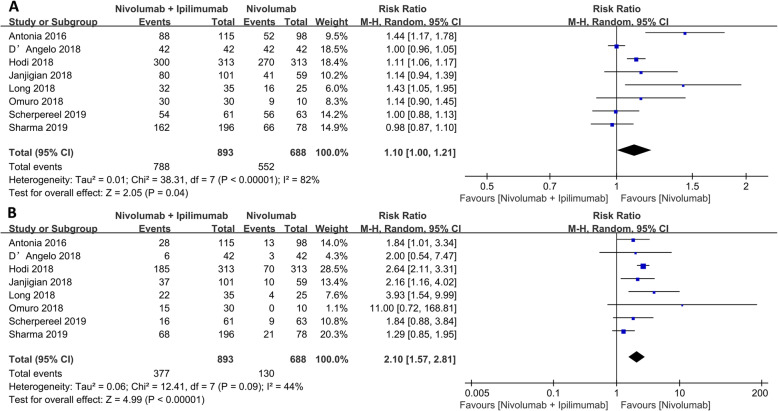
Forest plot of the adverse events between nivolumab combined with ipilimumab and nivolumab alone. **a** Any grade TRAEs. **b** Grade 3 or higher TRAEs

In terms of adverse effects, the incidence of any TRAEs and serious TRAEs were elevated in nivolumab monotherapy than in nivolumab plus ipilimumab group (RR: 1.10, 95% CI: 1.00–1.21, *p* = 0.04; RR: 2.10, 95% CI: 1.57–2.81, *p* < 0.001, respectively) as shown in Fig. 4.

### N1I3 versus N3I1

**Fig. 5:**
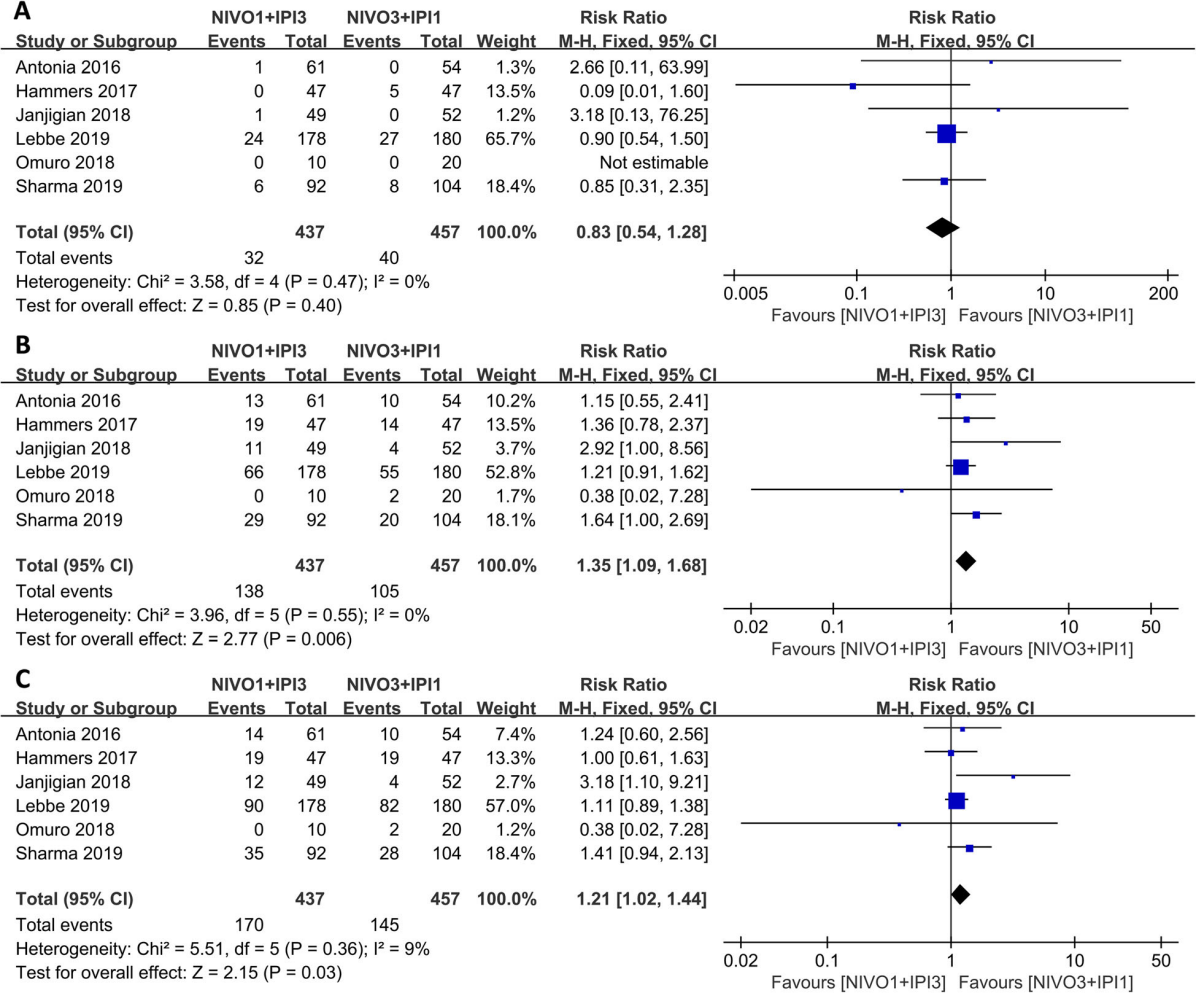
Forest plot of the overall effect of the nivolumab-ipilimumab group therapy (N1I3 versus N3I1). **a** Complete response (CR). **b** Partial response (PR). **c** Objective response rate (ORR)

**Fig. 6:**
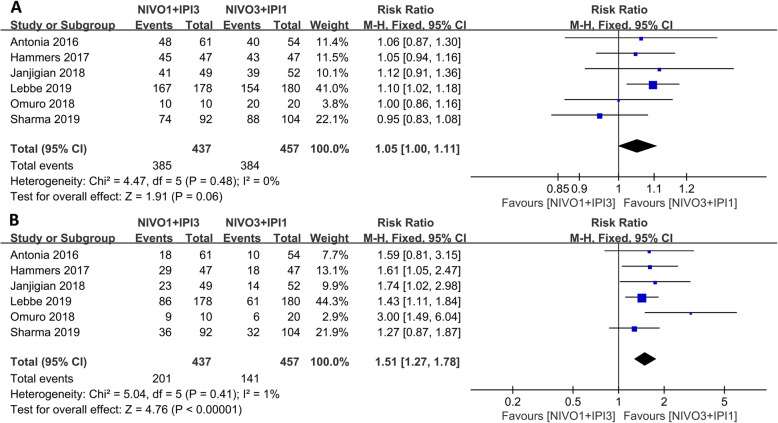
Forest plot of the adverse events of the nivolumab combined with ipilimumab (N1I3 versus N3I1). **a** Any grade TRAEs. **b** Grade 3 or higher TRAEs

Since the combined therapeutic effect was better than that of monotherapies, subgroup analysis of the combination therapy was further investigated. The N1I3 group showed better PR (RR: 1.35, 95% CI: 1.09–1.68, *p* = 0.006) and ORR (RR: 1.21, 95% CI: 1.02–1.44, *p* = 0.03), while there was no significant difference in CR (RR: 0.83, *p* = 0.40) between the two subgroups as shown in Fig. 5. There was no difference in TRAEs between the two groups as well (RR: 1.05, *p* = 0.06); however, N113 produced more serious adverse events (grade 3 or higher) than group N3I1 (RR: 1.51, 95% CI: 1.27–1.78, *p* < 0.001) as shown in Fig. 6).

Specific adverse treatment events by subgroup were also analyzed. Incidences of any grade adverse events were more elevated in group N1I3. These include increased alanine aminotransferase (ALT) (RR: 1.48, *p* = 0.02), increased aspartate aminotransferase (AST) (RR: 1.68, *p* = 0.004), diarrhea (RR: 1.47, *p* = 0.005), hypothyroidism (RR: 1.40, *p* = 0.04), and vomiting (RR: 1.77, *p* = 0.02). As shown in Table 1, adverse reactions, such as high ALT (RR: 2.25, *p* = 0.006) and diarrhea (RR: 2.90, *p* < 0.001), of grade 3 or above, were also high in group N1I3 than in group N3I1.

**Table 1:** Subgroup analysis of the treatment-related adverse events (TRAEs)

NIVO1 + IPI3 vs. NIVO3 + IPI1	No. of studies	RR	95% CI	*p*	Effect model	Heterogeneity	
						(*I*^2^)	*p*
Any grade increased ALT	6	1.48	1.06-2.06	*0.02*	Fixed	33%	0.19
Any grade increased AST	6	1.68	1.18-2.39	*0.004*	Fixed	41%	0.13
Any grade in pruritus	6	1.09	0.87-1.37	0.46	Fixed	0%	0.53
Any grade in diarrhea	6	1.47	1.18-1.83	*0.005*	Fixed	23%	0.26
Any grade in fatigue	6	1.06	0.88-1.29	0.53	Fixed	19%	0.29
Any grade in nausea	5	1.34	0.99-1.81	0.06	Fixed	0%	0.63
Any grade in hypothyroidism	5	1.40	1.01-1.94	*0.04*	Fixed	0%	0.78
Any grade in decreased appetite	5	1.16	0.81-1.64	0.42	Fixed	11%	0.35
Any grade in vomiting	4	1.77	1.11-2.84	*0.02*	Fixed	27%	0.25
Any grade in rash	6	1.29	0.98-1.70	0.07	Fixed	19%	0.29
Grade 3 or higher increased ALT	6	2.25	1.26-4.00	*0.006*	Fixed	0%	0.45
Grade 3 or higher increased AST	6	1.89	0.91-3.91	0.09	Fixed	12%	0.34
Grade 3 or higher in pruritus	6	0.82	0.22-3.10	0.77	Fixed	0%	0.53
Grade 3 or higher in diarrhea	6	2.90	1.63-5.15	*< 0.001*	Fixed	0%	0.85
Grade 3 or higher in fatigue	6	1.37	0.53-3.54	0.51	Fixed	19%	0.29
Grade 3 or higher in nausea	5	2.45	0.71-8.51	0.16	Fixed	0%	0.41
Grade 3 or higher in vomiting	4	1.63	0.39-6.79	0.50	Fixed	0%	0.51
Grade 3 or higher in rash	6	1.69	0.39-7.26	0.48	Fixed	0%	0.51

NIVO1 + IPI3, nivolumab 1 mg/kg plus ipilimumab 3 mg/kg, every 3 weeks for 4 doses (induction phase), followed by nivolumab 3 mg/kg, every 2 weeks until disease progression or unacceptable toxicity incidence of TRAEs (maintenance phase); NIVO3 + IPI1, nivolumab 3 mg/kg plus ipilimumab 1 mg/kg, every 3 weeks for 4 doses (induction phase), followed by nivolumab 3 mg/kg, every 2 weeks until disease progression or unacceptable toxicity incidence of TRAEs (maintenance phase)

Italic indicates that the results are statistically significant, and the *P*<0.05

## Discussion

Immunotherapy plays an important role in controlling tumors. Combination immunotherapy is based on the use of more than one immunotherapy. It can intervene and regulate multiple processes of the immune response through [30], chemoradiotherapy [31–33], and targeted therapy [34, 35] by promoting anti-tumor immune response reduce the risk of drug resistance. The combination of immunotherapies is one of the most promising approaches being studied [36]. In particular, the combination of anti-PD-1 (nivolumab) and anti-CTLA-4 (ipilimumab) has shown positive results in tumor treatment and significant enhancement in patients with metastatic melanoma [37], advanced RCC [38], and metastatic CRC [16, 39]. Cope et al. reported that recurrent SCLC showed better response to nivolumab plus ipilimumab compared to the current chemotherapy, interpreted as long-term survival benefits [40]. Ready et al. reported that the combination of nivolumab and low-dose ipilimumab was effective and tolerable as a first-line treatment of advanced/metastatic non-small cell lung cancer (NSCLC) [41]. Based on conditional survival analysis of first-line treatment, Shao [42] showed that patients with advanced RCC put on nivolumab plus ipilimumab therapy had a high survival rate compared with sunitinib.

Recently, the combination therapy of ipilimumab and anti-PD-1 antibody showed promising clinical benefit in some malignant tumors [43], advanced melanoma [44], RCC and other tumors [45]. Both combination therapy and nivolumab or ipilimumab monotherapy showed improved ORR, CR, and PR [38, 42, 46, 47]. The present systematic review showed that nivolumab plus ipilimumab group had significantly higher CR, PR, and ORR than ipilimumab monotherapy. CR with nivoluma plus ipilimumab therapy was 4.89 times higher than ipilimumab monotherapy, while the PR and ORR were 2.75 and 3.31 times than ipilimumab monotherapy, respectively. These findings show that the combination therapy was more effective than ipilimumab monotherapy. Elsewhere, Postow et al. [27] reported that the combination of nivolumab plus ipilimumab had a higher ORR and progression-free survival rate compared with ipilimumab monotherapy, in treatment-naive patients with advanced melanoma. Increased response rate and improved progression-free survival were reported in nivolumab plus ipilimumab group when compared with ipilimumab alone in a randomized phase III trial in treatment-naive patients with metastatic melanoma [48]. David et al. [49] compared the quality-adjusted survival rates of nivolumab plus ipilimumab group with ipilimumab monotherapy in untreated advanced melanoma patients. The result showed a combination group resulted in a statistically significant and clinically important improvement in quality-adjusted survival.

Nivolumab is a class of ICIs that PD-1 receptors that activate downstream signaling pathways by inducing FoxP3 expression [50] and promoting Treg (iTreg) cell differentiation [16]. The incidence of CR and PR and ORR in individuals on the combined immunotherapy group was 1.13, 1.50, and 1.40 times respectively, as high than those on nivolumab monotherapy. These findings emphasized the effectiveness of the combination therapy. Antonia et al .[19] reported that the combination of nivolumab plus ipilimumab had a higher prolonged anti-tumor activity in previously treated patients than nivolumab monotherapy. Preliminary data on metastatic RCC suggests that combination therapy had a higher ORR than nivolumab monotherapy in different trials [21, 51]. Morse [14] reported that a combination therapy (nivolumab with low-dose ipilimumab) had numerically higher response rates and improved long-term clinical benefit relative to anti-programmed death-1 monotherapy. Principally, CTLA-4 binds B7 ligand (B7-1/CD80 and B7-2/CD86) on antigen-presenting cells that compete with the CD28 receptor [16]. The CTLA-4 protein and its B7 ligand are mainly expressed on immune cells, suggesting that the CTLA-4 pathway plays a major role in lymph nodes. PD-L1, the PD-1 ligand, is widely expressed, mainly on regulatory peripheral T cell [52]. Although CTLA-4 and PD-1 antibodies are both checkpoint inhibitors, their action mechanisms are neither the same nor complementary [53]. Therefore, higher anti-tumor activity was seen with a combination therapy than ipilimulab or nivolumab [54].

Based on the above analysis, we can conclude that the combination of nivolumab plus ipilimumab is more effective than nivolumab alone, consistent with previous findings [19, 20]. Ghiringhelli et al. testified that checkpoint monotherapy inhibitors targeting PD-1 and PD-L1 were not effective in metastatic colorectal cancer patients with microsatellite stable tumors [55]. Neal compared nivolumab plus ipilimumab and nivolumab monotherapy in recurrent SCLC, whereas ORR was higher with nivolumab plus ipilimumab versus nivolumab, toxicities were more common with combination therapy versus nivolumab monotherapy [56]. However, what is different from the above research results is Kreft [57] showed that there was no difference in action and outcome between nivolumab plus ipilimumab group and nivolumab monotherapy in patients with melanoma.

Many combination immunotherapies have been developed, nivolumab plus ipilimumab being the most common [58]. There are two dosages of this combination: nivolumab 1 mg/kg plus ipilimumab 3 mg/kg (N1I3) and nivolumab 3 mg/kg plus ipilimumab 1 mg/kg (N3I1); however, no studies have been done to determine their differential effectiveness. Although there was no significant difference between N1I3 and N3I1 in CR, N1I3 yielded better resulted in PR and ORR than N3I1, suggesting that the efficacy of N1I3 may be better than that of N3I1. Sharma et al. [29] reported that with longer follow-up, N1I3 showed sustained antitumor activity than N3I1. Glutsch on his part investigated the presence and extent of side effects in advanced melanoma cases on nivolumab plus ipilimumab group. Here, renal toxicity was tolerable, and three doses of nivolumab (1 mg/kg) in combination with ipilimumab (3 mg/kg) showed deep partial relief on chest and abdominal CT scans [59]. This result not only supports our findings on N1I3, but emphasized on the potential benefit of combination immunotherapies in the tumor.

We also analyzed all common adverse reactions of every grade. Adverse reactions of grade 3 or higher in the combination group were elevated than those in monotherapy. Nivolumab plus ipilimumab group increased response rate with more side effects than ipilimumab monotherapy. Kreft [57] reported that nivolumab plus ipilimumab group was associated with a higher TRAEs compared with monotherapy, but N1I3 induced elevated grade 3 or higher TRAEs than N3I1, consistent with Antonia et al. [19]. Compared with N3I1, N1I3 improved the treatment benefit and slightly increased the incidence of TRAEs. What we need to make clear is that although N1I3 increased the rate of TRAEs, the overall incidence of TRAEs is controllable, and after symptomatic treatment, most of the conditions can be improved. When comparing with treatment groups, common grade 3 or 4 TRAEs in the nivolumab plus ipilimumab group arose early but resolved within the first 4–6 months of treatment [47]. Most selected treatment-related adverse events occurring within 30 days of the last dose in the nivolumab plus ipilimumab group were low-grade, and the majority resolved and were manageable using established algorithms [60].

The main TRAEs associated with nivolumab use include increased ALT and AST, pruritus, diarrhea, fatigue, nausea, hypothyroidism, decreased appetite, vomiting, and rash. After an extensive systematic review, Bajwa et al. found that the most common adverse effects encountered were colitis (14/139), hepatitis (11/139), adrenocorticotropic hormone insufficiency (12/139), hypothyroidism (7/139), type 1 diabetes (22/139), acute kidney injury (16/139), and myocarditis (10/139). The most common treatment approach was the cessation of the immune checkpoint inhibitor, initiation of steroids and supportive therapy [61, 62]. Motzer et al. reported that among all patients treated, the most common TRAEs in the grade 3-4 nivolumab plus ipilimumab group were elevated lipase (57 of 547 [10%]), elevated amylase (31 [6%]), and elevated ALT (28 [5%] )[47]. Reporting of corticosteroid use for ICIs has been effective among various studies.

There is an increasing number of immunotherapy and molecular targeting agents being evaluated in monotherapies as well as in various combinations, but the choice of right therapy, sequence, dosage of candidate agents, immunotherapies, and treatment for patients that progress on immune checkpoint inhibitors remains a challenge.

## Limitation

This meta-analysis only included four phase I clinical trials, which may reduce the credibility of the findings. In addition, this paper contains multiple tumor types, which may make the results of the study untargeted. It is necessary to point out that this paper was not able to extract hazard ratio (HR) as the effect size was insufficient, but this is the first meta-analysis to compare nivolumab plus ipilimumab with ipilimumab or nivolumab monotherapy.

## Conclusions

This paper showed that the curative effect of nivolumab plus ipilimumab therapy is better than ipilimumab or nivolumab monotherapy. In the combination group, N1I3 is more effective than N3I1. Although side effects were slightly increased in group N1I3, the overall safety was reliable.

## Supplementary information

**Additional file 1: Supplementary Table. 1.** Characteristics of included clinical trials in the meta-analysis.

**Additional file 2: Supplementary Figure 1.** Flow chart of study selection.

## Data Availability

All data are available within the article.
