# Supplementary material for: The efficacy and safety of combined immune checkpoint inhibitors (nivolumab plus ipilimumab): a systematic review and meta-analysis
Source: World J Surg Oncol. 2020 Jul 3;18:150. doi: 10.1186/s12957-020-01933-5 (PMC7334852; doi:10.1186/s12957-020-01933-5)
Supplement: Supplementary file 1 — Additional file 1: Supplementary Table. 1. Characteristics of included clinical trials in the meta-analysis. [file 12957_2020_1933_MOESM1_ESM.docx]

**Supplementary Table 1**. Characteristics of included clinical trials in the meta-analysis

| Author,  year | Phase | Tumor | Therapeutic regimen | | |
| --- | --- | --- | --- | --- | --- |
|  |  |  | Nivolumab + Ipilimumab | Nivolumab alone | Ipilimumab alone |
| Antonia,  2016 | 1/2 | SCLC | nivolumab(1mg/kg)+ipilimumab(3mg/kg) Q3W 4 doses(induction phase);  nivolumab(3mg/kg) Q2W (maintenance phase)  nivolumab(3mg/kg)+ipilimumab(1mg/kg) Q3W 4 doses(induction phase);  nivolumab(3mg/kg) Q2W (maintenance phase) | nivolumab(3mg/kg) Q2W |  |
| D^’^Angelo,  2018 | 2 | Sarcoma | nivolumab(3mg/kg)+ipilimumab(1mg/kg) Q3W 4 doses(induction phase);  nivolumab(3mg/kg) Q2W (maintenance phase) | nivolumab(3mg/kg) Q2W |  |
| Hammer,  2017 | 1 | RCC | nivolumab(1mg/kg)+ipilimumab(3mg/kg) Q3W 4 doses(induction phase);  nivolumab(3mg/kg) Q2W (maintenance phase)  nivolumab(3mg/kg)+ipilimumab(1mg/kg) Q3W 4 doses(induction phase);  nivolumab(3mg/kg) Q2W (maintenance phase) |  |  |
| Hodi,  2018 | 3 | Melanoma | nivolumab(1mg/kg)+ipilimumab(3mg/kg) Q3W 4 doses(induction phase);  nivolumab(3mg/kg) Q2W (maintenance phase) | nivolumab(3mg/kg) Q2W | ipilimumab(3mg/kg) Q3W |
| Janjigian,  2018 | 3 | EGC | nivolumab(1mg/kg)+ipilimumab(3mg/kg) Q3W 4 doses(induction phase);  nivolumab(3mg/kg) Q2W (maintenance phase)  nivolumab(3mg/kg)+ipilimumab(1mg/kg) Q3W 4 doses(induction phase);  nivolumab(3mg/kg) Q2W (maintenance phase) | nivolumab(3mg/kg) Q2W |  |
| Lebbe,  2019 | 3/4 | Melanoma | nivolumab(1mg/kg)+ipilimumab(3mg/kg) Q3W 4 doses(induction phase);  nivolumab(480mg) Q4W (maintenance phase)  nivolumab(3mg/kg)+ipilimumab(1mg/kg) Q3W 4 doses(induction phase);  nivolumab(480mg) Q4W (maintenance phase) |  |  |
| Long,  2018 | 2 | Melanoma | nivolumab(1mg/kg)+ipilimumab(3mg/kg) Q3W 4 doses(induction phase);  nivolumab(3mg/kg) Q2W (maintenance phase) | nivolumab(3mg/kg) Q2W |  |
| Omuro,  2018 | 1 | Glioblastoma | nivolumab(1mg/kg)+ipilimumab(3mg/kg) Q3W 4 doses(induction phase);  nivolumab(3mg/kg) Q2W (maintenance phase)  nivolumab(3mg/kg)+ipilimumab(1mg/kg) Q3W 4 doses(induction phase);  nivolumab(3mg/kg) Q2W (maintenance phase) | nivolumab(3mg/kg) Q2W |  |
| Postow,  2017 | 2 | Melanoma | nivolumab(1mg/kg)+ipilimumab(3mg/kg) Q3W 4 doses(induction phase);  nivolumab(3mg/kg) Q2W (maintenance phase) |  | ipilimumab(3mg/kg) Q3W |
| Scherpereel,2019 | 2 | MPM | nivolumab(3mg/kg) Q2W + ipilimumab(1mg/kg) Q6W | nivolumab(3mg/kg) Q2W |  |
| [Sharma](https://www.ncbi.nlm.nih.gov/pubmed/?term=Sharma%20P%5bAuthor%5d&cauthor=true&cauthor_uid=31100038),  2019 | 1/2 | Urothelial carcinoma | nivolumab(1mg/kg)+ipilimumab(3mg/kg) Q3W 4 doses(induction phase);  nivolumab(3mg/kg) Q2W (maintenance phase)  nivolumab(3mg/kg)+ipilimumab(1mg/kg) Q3W 4 doses(induction phase);  nivolumab(3mg/kg) Q2W (maintenance phase) | nivolumab(3mg/kg) Q2W |  |

SCLC, small cell lung cancer; RCC, Renal Cell Carcinoma; EGC, Esophagogastric Cancer; MPM, Malignant pleural mesothelioma.
