# Supplementary figures and images for: The efficacy and safety of combined immune checkpoint inhibitors (nivolumab plus ipilimumab): a systematic review and meta-analysis
Source: World J Surg Oncol. 2020 Jul 3;18:150. doi: 10.1186/s12957-020-01933-5 (PMC7334852; doi:10.1186/s12957-020-01933-5)

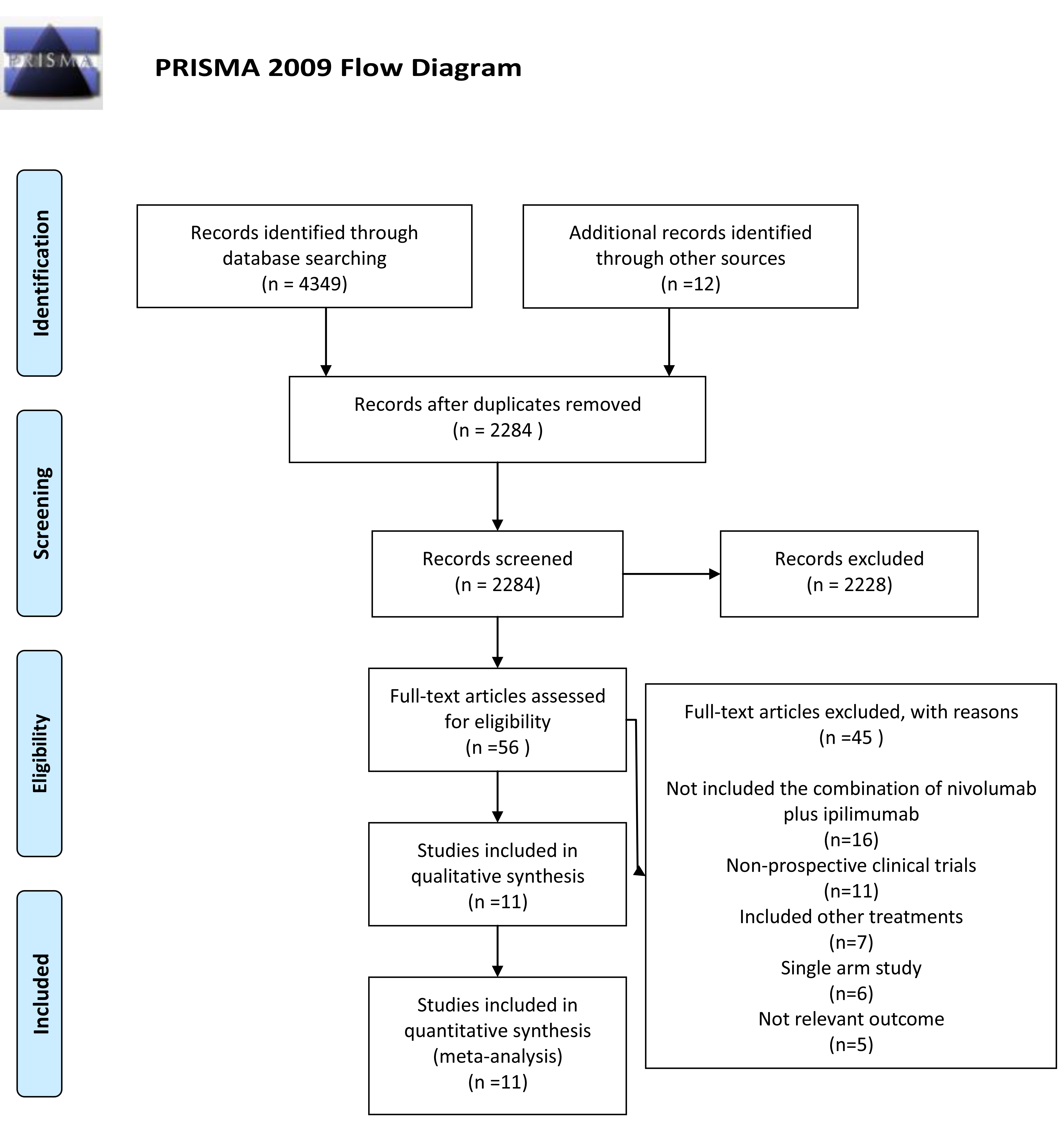

Supplement: Supplementary file 2 — Additional file 2: Supplementary Figure 1. Flow chart of study selection. [file 12957_2020_1933_MOESM2_ESM.tif]
